# Exploring Self-Paced Embodiable Neurofeedback for Post-stroke Motor Rehabilitation

**DOI:** 10.3389/fnhum.2019.00461

**Published:** 2020-01-20

**Authors:** Nadine Spychala, Stefan Debener, Edith Bongartz, Helge H. O. Müller, Jeremy D. Thorne, Alexandra Philipsen, Niclas Braun

**Affiliations:** ^1^Neuropsychology Lab, Department of Psychology, University of Oldenburg, Oldenburg, Germany; ^2^Department of Psychiatry and Psychotherapy, University of Bonn, Bonn, Germany

**Keywords:** neurofeedback, motor imagery, brain computer interface, sense of ownership, sense of agency, stroke, rubber hand illusion

## Abstract

Neurofeedback-guided motor-imagery training (NF-MIT) has been proposed as a promising intervention following upper limb motor impairment. In this intervention, paretic stroke patients receive online feedback about their brain activity while conducting a motor-imagery (MI) task with the paretic limb. Typically, the feedback provided in NF-MIT protocols is an abstract visual signal based on a fixed trial. Here we developed a self-paced NF-MIT paradigm with an embodiable feedback signal (EFS), which was designed to resemble the content of the mental act as closely as possible. To this end, the feedback was delivered *via* an embodiable, anthropomorphic robotic hand (RH), which was integrated into a closed-looped EEG-based brain-computer interface (BCI). Whenever the BCI identified a new instance of a hand-flexion or hand-extension imagination by the participant, the RH carried out the corresponding movement with minimum delay. Nine stroke patients and nine healthy participants were instructed to control RH movements as accurately as possible, using mental activity alone. We evaluated the general feasibility of our paradigm on electrophysiological, subjective and performance levels. Regarding electrophysiological measures, individuals showed the predicted event-related desynchronization (ERD) patterns over sensorimotor brain areas. On the subjective level, we found that most individuals integrated the RH into their body scheme. With respect to RH control, none of our participants achieved a high level of control, but most managed to control the RH actions to some degree. Importantly, patients and controls achieved similar performance levels. The results support the view that self-paced embodiable NF-MIT is feasible for stroke patients and can complement classical NF-MIT.

## Introduction

Motor impairments in the upper limbs are among the most prevalent symptoms following stroke (Grefkes and Ward, 2014). Neurofeedback-guided motor imagery training (NF-MIT) has been proposed as a promising intervention for treating upper limb motor impairments (Pichiorri et al., 2015; Zich et al., 2017). In this intervention, paretic stroke patients conduct a motor imagery (MI) task during which they receive online neurofeedback about their brain activity. The therapeutic idea behind NF-MIT is to provide feedback to the patients on how well they are performing, by showing a beneficial neuronal activation pattern thought to support motor recovery (Sitaram et al., 2017). Typically, the feedback is a rather abstract signal, such as a moving bar or ball presented on a computer screen. In the context of an embodied cognition view (Wilson, 2002; Foglia and Wilson, 2013; Wilson and Golonka, 2013), it can be argued that an embodiable feedback signal (EFS) is more natural and intuitive for the patient. A feedback signal that closely resembles the MI act performed, in both time and space, may be potentially better accepted by patients, in particular if they suffer from cognitive impairments, and may, eventually, lead to better performance.

A few studies have developed an EFS (Perez-Marcos et al., 2009; Alimardani et al., 2013; Ono et al., 2013; Pichiorri et al., 2015; Braun et al., 2016). Most of these studies were inspired by the active rubber hand illusion (aRHI; Kalckert and Ehrsson, 2012, 2014; Braun et al., 2014) or its VR-based derivatives (Slater et al., 2008, 2009; Sanchez-Vives et al., 2010; Kilteni et al., 2012; Ma and Hommel, 2013; Pichiorri et al., 2015; Ma et al., 2017). The aRHI is a special variant of the classical rubber hand illusion, in which a movable artificial robotic hand (RH), rather than a static rubber hand, is placed visibly, and in an anatomically-plausible position, in front of the individual, while the individual’s own hand is hidden from view. If the RH is repeatedly moved in synchrony with the individual’s real or imagined hand movements, an illusory sense of ownership (SoO) and sense of agency (SoA) for the artificial hand can typically be induced (Kalckert and Ehrsson, 2012, 2014; Braun et al., 2014, 2016). That is, individuals may then experience the RH as part of their own body (SoO) and its movements as under their voluntary movement control (SoA). In order to provide real-time feedback for the MI within an aRHI paradigm, imagined hand movements are decoded from electrical brain activity and the corresponding commands are issued to the RH. Ideally, the RH then executes the imagined movements with little temporal delay (Braun et al., 2016).

Several studies have indicated beneficial effects of EFS (Perez-Marcos et al., 2009; Slater et al., 2009; Alimardani et al., 2013, 2014). Ono et al. (2013), for instance, compared different feedback signals presented on a computer screen and found evidence for a more robust event-related desynchronization (ERD) pattern for natural as compared to abstract feedback signals. Also, in a previous aRHI study (Braun et al., 2016), we used a RH and investigated the role of feedback-signal embodiment. Individuals experienced RH movements as more self-related and self-caused if the RH was placed in a congruent position such that it could be embodied. Individuals were also able to induce RH movements more quickly. These findings suggest that natural feedback signals may help to improve the quality of NF-MIT. It is not clear, however, to what extent stroke patients in particular benefit from an EFS, since most paradigms have been tested only with healthy individuals. A recent meta-analysis conducted by Cervera et al. (2018) showed that brain-computer interface (BCI)-based neurorehabilitation on upper-limb motor function can lead to more improvement in motor performance than other conventional therapies, supporting the general effectiveness of classical NF-MIT in stroke patients. The specific effect of EFS, however, has not yet been studied.

Most NF-MIT paradigms employ cue-based designs, in which the timing and content of mental tasks are predefined by visual or auditory cues (Scherer et al., 2008). Such rigid training regimes are easy to control experimentally, but they suffer from poor ecological validity, in particular when voluntary actions are studied. Spontaneous, self-paced designs may be needed to allow individuals to develop self-control and increased acceptance (Lotte et al., 2013).

In the present study, we investigated whether a self-paced embodiable NF-MIT, in which the individuals can freely explore the consequences of their different MI acts on a RH, is feasible. We tested this RH neurofeedback paradigm with stroke patients (*n* = 9), since this is the major target group for which NF-MIT is ultimately intended, as well as with healthy participants matched in age and gender. We wanted to know whether stroke patients, in particular, are able to learn to control the RH. To evaluate neurofeedback performances, participants had to perform various tasks such as conducting as many RH movements as possible in some time periods and withholding any RH movements in others. Behavioral, subjective and electrophysiological measures were collected and analyzed.

## Materials and Methods

### Participants

Nine chronic stroke patients (one female) aged 55–75 and nine healthy controls matched in age and sex were recruited for the study (see Table 1, for demographic and clinical data). All participants were required to have a normal or corrected-to-normal vision and no known history of a mental disorder. All patients suffered from a first-time monolateral stroke. Months since stroke ranged from 15 to 72 months, *M* = 41.44, *SD* = 22.6. Inclusion criterion was a moderate to severe right-hand paresis due to the stroke as assessed by the Fugl-Meyer test (see “Stroke Patient Assessment” section). Patients were required to have no dementia, no epileptic seizures, and no severe neglect or severe aphasia that would impair their ability to follow task instructions. All participants were compensated for their participation (8€ per hour), gave written informed consent and were naive to the purpose of the study. The study was approved by the University of Oldenburg ethics committee.

**Table 1:** Demographic data of stroke patients and healthy controls.

Characteristics	Stroke (*N* = 9)	Control (*N* = 9)
Sex (male: female)	8:1	8:1
Age (SD)	60.33 (9.31)	60.22 (9.77)
Motor assessment (SD)	27.88 (15.21)	-
Sensibility assessment (SD)	34.00 (4.38)	-
Infarct side (left: right: both)	8:0:1	-
Infarct location (cortical: subcortical: mixed)	3:4:2	-
MOCA (SD)	21.77 (2.58)	-

### Overview

The study was carried out over three sessions for stroke patients and two sessions for healthy controls. The additional session for the stroke patients served to conduct the cognitive, motor and sensory assessments. The other two sessions, in which the actual NF-MIT was conducted were identical for both groups. Here, we report NF-MIT data from the second of these two sessions. In the first session, participants had to kinesthetically imagine flexion/extension movements with both of their hands in spatiotemporal synchrony with the RHs flexion/extension movements, while in the second session, they imagined these movements with only their right hand. Thus, the first session implemented a different experimental task and will be reported elsewhere.

### Stroke Patient Assessment

Cognitive assessment was conducted using the current version of the Montreal cognitive assessment (MoCA; Nasreddine et al., 2005). This test covers different cognitive domains and is an established fast screening tool for detecting mild cognitive impairments. The MoCA score ranges from 0 to 30 and German-speaking participants with normal cognitive ability are expected to exceed a threshold between 18 and 24 (depending on age and gender; Thomann et al., 2018).

Motor assessment was carried out using an adapted version of the Fugl-Meyer test (Sanford et al., 1993). While the original Fugl-Meyer test assesses both upper and lower limb movements, we only focused on 29 upper-limb tasks in the present study. All movement tasks were first executed with the non-paretic and then with the paretic arm. For each task, the achieved motor performance scores of the paretic and the non-paretic arm were compared and their difference assessed on a 3-point Likert scale. The scale ranged from zero (clearly lower performance on the paretic side) to two (identical performance on the paretic and non-paretic side). A summation score was then calculated by adding up all 29 individual task scores. The maximum score achievable was thus 58. The cut-off criterion indicating mild to severe right-hand paresis was set to scores lower than, or equal to, 45.

The sensory assessment was based on a testing procedure adapted from the Nottingham Sensory Assessment (Lincoln et al., 1998). We tested six different sensory modalities (pressure, light touch, pain, temperature, proprioception and vibration) on three different upper limb locations (upper arm, forearm, back of the hand), while the patients’ eyes were closed. For each modality and each position, sensibility performances of the paretic and the non-paretic body side were compared and assessed on a 3-point Likert scale, ranging from zero (no detection of respective sensory stimulus), to two (identical sensory detection performance on the paretic and the non-paretic side). A summation score was then calculated by adding up all 18 individual task scores. The maximum score achievable was thus 36.

### Apparatus

**Figure 1:**
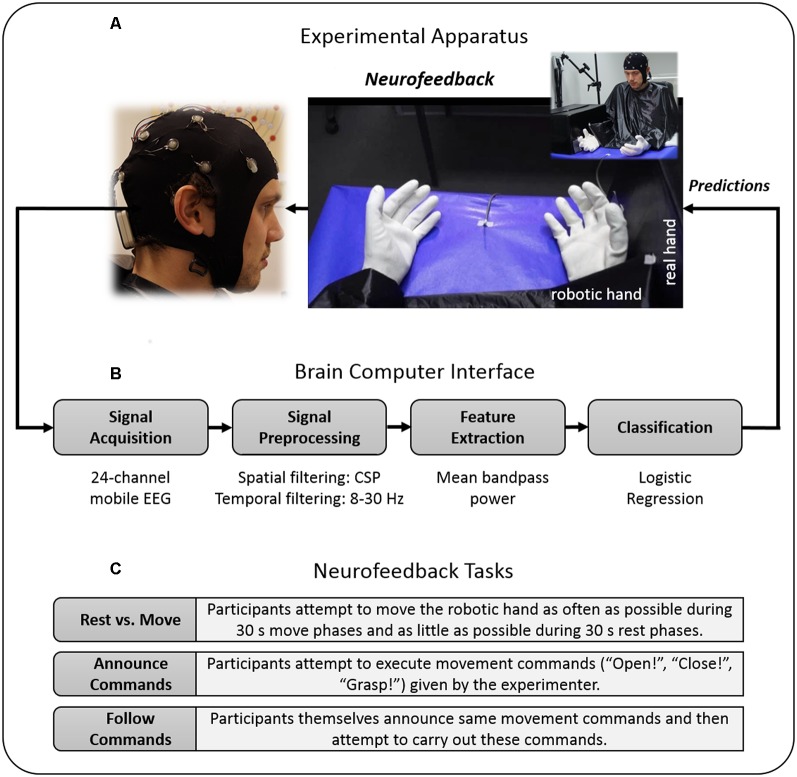
Study design. **(A)** Experimental apparatus. Participants placed their right hand into a black box, whereas the robotic hand (RH) was placed directly alongside in front of the participant. During the training block, participants kinesthetically imagined flexion and extension movements in spatio-temporal synchrony to the flexion and extension movements of the RH. **(B)** Brain-Computer Interface (BCI). For neurofeedback provision, the RH was connected to a BCI that was trained on the training block data and moved whenever the BCI detected the imagination of either a participant’s flexion or extension. The BCI’s classification algorithm was based on signal features of the 8–30 Hz sensorimotor rhythm (SMR). **(C)** Neurofeedback tasks. During the feedback block, participants attempted to control the RH’s movements as accurately as possible, using their motor-imagery (MI) thoughts alone. To evaluate their achieved controllability over the RH, participants had to perform different “BCI Parcours” in which they attempted to carry out various computer-given, experimenter-given or self-given commands. Data privacy remark: The person shown on the figure agreed with the publication of this figure.

The NF-MIT took place in a sound-attenuated and dimly lit experiment room. The experimental setting was adapted from our previous study (Braun et al., 2016) and is depicted in Figure 1A. The participant sat in front of a rectangular table (50 × 60 cm) and placed the right hand into a black box. This box was upholstered on the inside, so as to allow for a comfortable placement, and covered both hand and lower arm, hiding them from view. The anthropomorphic RH was placed in an anatomically-congruent position next to the black box, such that it was positioned medially aside the hidden real right hand. The horizontal distance between the participant’s real right hand and RH was around 7.5 cm. A green LED was placed in the middle of the table. Both the RH and the participant’s right (unseen) hand were covered with a thin-gauge garden glove. A blanket covered shoulders and arm and the space between the RH and the participant’s body. The aim of this was to facilitate the visual impression that the RH could be the participant’s own hand. The RH closely resembled a typical large human hand in terms of shape and size, and could be controlled with Matlab R2012a (Mathworks, Natick, MA, USA) *via* a microcontroller (Arduino mega 2560). The RH realistically mimicked hand flexion and hand extension movements (for more details, see Braun et al., 2016). The time delay between a Matlab control command sent to the microcontroller and an actual RH movement onset was less than 200 ms. The NF-MIT itself consisted of two blocks, a training block, and a feedback block.

### Neurofeedback Motor-Imagery Training

#### Training Block

The training block served to acquaint the participants with the MI task and to calibrate the classifiers used in the ensemble classification algorithm for the ensuing feedback block (see “EEG Data Recording” and “EEG Data Analysis and Classifier Training” sections for more detail). To enable a successful aRHI induction in the feedback block, RH movements were already included in the training. The training block lasted 12 min and was based on a fixed trial structure, and consisted of around 50 runs, each run lasting around 14 s and beginning with a 5 s rest period. During the initial rest period, the RH was in the open state and participants were instructed to relax and not to move. After that, a small LED indicated to the participants to prepare for the subsequent MI flexion trial, which began 300 ms after LED onset and lasted for 1.5 s. During this period, the RH flexed its fingers and the participants were required to concomitantly imagine the same movement with their right hand. The instruction was thus to kinesthetically imagine the same flexion movement, in spatiotemporal synchrony with the RH movement. The flexion period was finished by the offset of the LED and the participants were instructed to relax again for another 5 s. Then, the LED switched on again, preparing the participants for the extension trial, which began 300 ms after LED onset. The extension trial also lasted 1.5 s, during which the RH extended its fingers and participants were required to concomitantly imagine this extension movement with their right hand. The extension trial was finished by the offset of the LED and the next run began.

#### Neurofeedback Block

During the neurofeedback block, the RH was connected to a BCI that was trained on the training block data (see “EEG Data Recording” section), and moved whenever the BCI’s classification algorithm assumed an instance of flexion or extension imagination. The neurofeedback block consisted of an acquaintance phase and three neurofeedback tasks (Figure 1C), each of which lasted 4 min.

During the acquaintance phase, the participants had the opportunity to familiarize themselves with self-paced NF-MIT. That is, they were allowed to freely try different MI acts and to observe how these acts influenced the RH’s movement behavior. The overall aim thereby was to gain as much control as possible over the RH’s flexion, extension, and resting states, using MI thoughts alone. To help the participants to accomplish this aim, we suggested they try different mental strategies (e.g., “If you want to flex the RH, try to imagine grasping something or recall the flexion imagination from the training phase” or “If you want to rest the RH, mentally count numbers”). Moreover, the participants were asked whether some threshold modifications within the classification algorithm needed to be done; for instance, whether they remained trapped within one RH state. The classifier thresholds of the logistic regression used in the ensemble classification algorithm were then adjusted according to which thresholds best enabled participants to control the RH using MI. That is, if for instance the RH opened more often than intended by the participant, we set the threshold of the respective classifier to a higher value, e.g., from 0.7 to 0.8 (see “EEG Data Analysis and Classifier Training” and “Ensemble Classification Algorithm and Online Data Flow” sections).

The first neurofeedback task was a “rest vs. move task,” during which the participants attempted to move the RH as often as possible during 30 s move phases and as little as possible during 30 s rest phases. The respective phases alternated eight times and were indicated by a small LED that switched on during move phases and switched off during rest phases.

The second neurofeedback task was a “follow commands task,” during which the participants attempted to carry out movement commands given by the experimenter. To this end, the experimenter sat next to the participants, equipped with a laptop, and time-marked each given command. The experimenter chose one from four different commands, three explicit commands and one implicit commands. The explicit commands were “open,” “close” and “grasp,” where grasp meant to open (extension) and immediately close (flexion) the RH. The implicit command was to keep the RH in its momentary resting state (i.e., either “remaining opened” or “remaining closed”), as long as the experimenter gave no new explicit command.

The third neurofeedback task, the “announce commands task,” was identical to the follow commands tasks, with the exception that this time the participants themselves announced their next intended RH movement. Participants orally communicated the commands to the experimenter and thereafter aimed to initiate the announced movement. Commands were again time-marked by the experimenter. During the times in which the participant gave no command it was assumed that the participant currently did not intend to move the RH. Further, during the times in which the participant announced a command, but had not yet achieved the announced movement, it was assumed that the participant was still attempting to carry out the announced movement.

### EEG Data Recording

EEG data acquisition was done with a mobile, 24-channel EEG system (mBrainTrain GmbH, Belgrad, Serbia) using an elastic cap (EASYCAP, Herrsching, Germany). The cap’s electrode montage was a subset of the 10–20 system. It included the following positions: FP1, FP2, F7, F8, FZ, FC1, FC2, T7, C3, CZ, C4, T8, TP9, CP5, CP1, CPz, CP2, CP6, TP10, P3, PZ, P4, O1, and O2. AFz served as ground (DRL) and FCz as reference (CMS). The continuous EEG signal was digitized *via* Lab Streaming Layer (LSL^1^) with a sampling rate of 500 Hz and 24-bit resolution.

### EEG Data Analysis and Classifier Training

To prepare the neurofeedback, EEG training data were analyzed on-site with EEGLAB (Delorme and Makeig, 2004) and BCILAB (Kothe and Makeig, 2013). More specifically, three probabilistic classifiers were derived and used as the basis for an ensemble-like classification algorithm (see details below). A first classifier was calibrated for discriminating flexion trials from extension trials, a second for discriminating flexion trials from rest trials, and a third for discriminating extension trials from rest trials. That is, the first classifier output referred to the probability of flexion as opposed to the extension, the second to the probability of flexion as opposed to remaining open, and the third to the probability of extension as opposed to remaining closed. The three classifiers were trained on different time segments but otherwise underwent the same signal processing steps. To derive each classifier, the EEG training data were first band-pass filtered from 8 to 28 Hz and then epoched into 1.5 s segments relative to the onsets of the extension, flexion, and rest trials. In total there were thus twice as many rest trials as extension or flexion trials. Next, the segments that included obvious non-stereotyped artifacts were identified and rejected using built-in EEGLAB functions (Delorme et al., 2007). The remaining segments were submitted to an adaptation of BCILAB’s pre-built ParadigmCSP class (Kothe and Makeig, 2013). This paradigm detects class-specific changes in the sensorimotor rhythm (SMR) by means of common spatial pattern (CSP) analysis. Briefly described, given two time windows of a multivariate signal, CSP finds spatial filters that minimize the variance for one class and simultaneously maximize the variance for the other class (for reviews, see Ramoser et al., 2000; Blankertz et al., 2008). Using ParadigmCSP, 24 CSP filters were derived for each classifier. From these 24 spatial filters, the first four and last four filters (i.e., those filters who promised the highest class-discriminability) were inspected with respect to their spatial topography and associated time course. All CSP-filters showing physiologically-plausible sensorimotor cortex activity were kept, and feature values were calculated by multiplying each EEG segment with each CSP-filter and then taking the log-variance from the CSP segments. For each classifier, the feature space was equal to the number of CSP filters used. To obtain probabilistic class estimates, a regularized logistic regression model as implemented in BCILAB was then trained on each of the three derived training sets (feature matrices). Given a training set of observations whose class membership is known, this machine learning algorithm learns to make probability estimates on the class membership of new observations (Dreiseitl and Ohno-Machado, 2002). These probability estimates served as the basis for our ensemble-like classification algorithm described below.

### Ensemble Classification Algorithm and Online Data Flow

**Figure 2:**
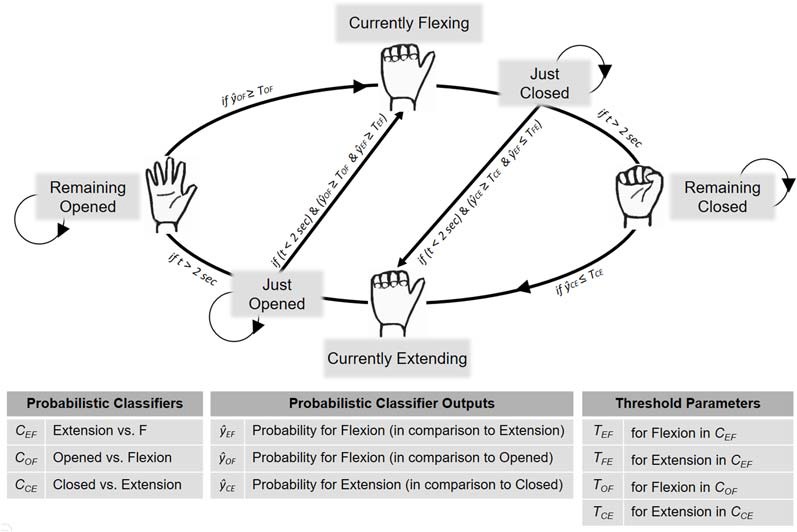
BCI implementation for neurofeedback provision. To control the RH’s movements and resting states during the neurofeedback block, an ensemble-like classification algorithm was implemented, in which the ultimate classifier output depended on the situation-dependent combination of three individual classifiers (${\hat{Y}}_{\text{EF}}$, ${\hat{Y}}_{\text{OF}}$, ${\hat{Y}}_{\text{CE}}$). That is, the RH’s current state (which was either “Remaining Open,” “Currently Flexing,” “Just Closed,” “Remaining Closed,” “Currently Extending” or “Just Opened”) determined how the three estimates were combined to derive the final classification algorithm’s decision. Further details are provided in the main text.

To deliver the feedback, Matlab, LSL and BCILAB were used. The data processing and feature extraction followed the same procedure as during classifier training (Figure 1B). The classifiers always operated on the most current 1.5 s. EEG segment and were constantly updated every 50 ms. To control the RH’s movements and resting states, an ensemble-like classification algorithm was implemented, in which the ultimate classifier output depended on the weighted estimates of the three individual classifiers. The usage of the three classifiers’ estimates was determined by the RH’s current state, being one of six different states: “remaining opened,” “currently flexing,” “just closed,” “remaining closed,” “currently extending” and “just opened” (Figure 2). For an illustration of one cycle of the RH’s state transitions, first, assume that the RH is in the “remaining opened” state. In that state only the open vs. flexion classifier is active and the RH remains in this state until the constantly-updated classifier output reaches a certain threshold (e.g., *p* = 0.7 for the flexion class), individually adjusted for the participant during the acquaintance phase. Once a threshold is reached, the RH starts flexing, i.e., it switches into the “currently flexing” state. During the “currently flexing” state, no classifier is active and the RH remains in this state for as long as the flexion movement is being completed (2 s). Next, the RH switches into the “just closed” state, during which the closed vs. extension classifier and the extension vs. flexion classifier are active. The RH remains in this state either until both classifier outputs reach their respective thresholds, or until 2 s have passed. In the first case, the classification algorithm assumes an extension has been imagined and thus immediately induces an extension movement, that is, it switches into the “currently extending” state. In the latter case, the RH switches into the “remaining closed” state, during which only the closed vs. extension classifier is active. The RH remains in this state until the closed vs. extension classifier output reaches its defined threshold and then it starts extending, that is, it switches into the “currently extending” state. During the “currently extending” state, no classifier is active and the RH remains in this state until the extension movement is completed (2 s). Next, the RH switches into the “just opened” state, during which the open vs. flexion classifier and the extension vs. flexion classifier are active. The RH remains in this state either until both classifier outputs meet their defined thresholds, or until 2 s have passed. In the first case, the classification algorithm assumes a flexion has been imagined and thus immediately induces a flexion movement, that is, it switches into the “currently flexing” state. In the latter case, the RH switches into the “remaining open” state.

### EEG Offline Analysis

#### Time-Frequency Analysis

MI-induced changes in the SMR served as the physiological basis for our classification algorithm. To further explore these oscillatory changes, an offline EEG time-frequency (TF) analysis was carried out on the training block data. EEG artifacts were attenuated using extended infomax independent component analysis (ICA; Bell and Sejnowski, 1995; Delorme and Makeig, 2004). Artifactual independent components were identified by visual inspection and excluded from the back projection. Next, the ICA-corrected continuous data were segmented from −2 to 3 s, relative to the onsets of the MI flexion and MI extension trials. EEG segments containing unique, non-stereotyped artifacts were identified by built-in EEGLAB functions and rejected. From each remaining EEG segment, a corresponding CSP segment was calculated by multiplying the data with a chosen CSP-filter. To obtain a unique CSP-component for each participant, all 24 potential CSP components were first derived from each segment and then the physiologically most plausible CSP component was kept. A TF analysis was carried out on the derived CSP segments by means of a continuous Morlet wavelet transform (Debener et al., 2005; Thorne et al., 2011). The hereby obtained frequency bins ranged from 5 to 50 Hz in 1 Hz frequency steps. The TF analysis was conducted for the time interval from −0.8 to 2.3 s.

To avoid edge artifacts, TF data were only analyzed from −0.5 s to 2 s, relative to the beginning of MI. Percent power change relative to baseline power was calculated. That is, for each frequency bin, the corresponding time series was first squared, then scaled to decibels (10 × log10) and finally its change in power (relative to the first 0.5 s mean baseline power) was calculated. For the statistical analysis, SMR power changes were separately extracted for flexion and extension trials. To this end, across trials, mean percent log power changes between 10 and 25 Hz were calculated for the 1 s time interval beginning 0.5 s after MI onset.

#### ERD Latency Analysis

Based on the TF data, an ERD latency value was calculated, reflecting the time intervals between MI period onsets and ERD onsets. To this end, a threshold defined as a power reduction of at least 30% as a corresponding desynchronization was set. To counterbalance outliers, the 20th percentile of all trials exceeding the determined threshold was taken to calculate a mean ERD latency across trials for each participant.

#### CSP Filter Analysis

To further evaluate the neurophysiological basis of our NF-MIT, we investigated the quality of the CSP filters that were calculated for the online analysis. For this purpose, six criteria were extracted from the heuristics of appropriate filter selection (see Supplementary Material S1). These criteria were, first, whether the signal in the CSP pattern appeared to originate from the sensorimotor areas; second, whether the signal in the CSP filter appeared to originate from the sensorimotor areas; third, whether there was a recognizable discriminability between the compared trial classes in the power value distributions; fourth, whether there was recognizable discriminability between trial classes on single-trial time course visualizations; fifth, whether a left-sided ERD and/or a right-sided ERD or ERS occurred; and finally, whether the power value distributions for each trial class were normally distributed. Two CSP filters (one for flexion, one for extension) for each participant were evaluated on these criteria. Each criterion could be either fulfilled or not. A sum value across the two CSP filters was calculated, leading to a maximum score of 12 points. We defined that scores ≥10 indicated plausible CSP filters.

### Questionnaire Data

A 15-item questionnaire (see Table 2), adapted from previous studies (Kalckert and Ehrsson, 2012; Braun et al., 2014, 2016), was used to assess the participant’s subjective experiences. At the end of each block, the experimenter read each item to the participant, and the participant had to rate his or her level of agreement on a 7-point Likert scale. The scale ranged from −3 (“totally disagree”) to +3 (“totally agree”). SoO, SoA and two other phenomenal target properties—experiential realness (ER) and MI-action binding (MIAB)—were operationalized. SoA was defined as the amount of experienced authorship over the RH’s movement behavior and SoO as the experienced level of “mineness” towards the RH. MIAB indicated the extent to which the self-induced MI percept and the RH motion percept felt bound together and ER the extent to which the MI act was felt as real and vivid. Three items were used for each phenomenal target property and later averaged to obtain a single value for each block. The remaining three items were control items, one relating to SoA, and two relating to SoO. These items entailed illusion-related statements but did not specifically capture the phenomenal experience of limb ownership or SoA. Hence, in the case of a successful SoO and SoA induction, items related to these two phenomenal constructs should have high affirmative ratings, whereas the control items should not be specifically affected by the experimental manipulation. As in former studies (Kalckert and Ehrsson, 2012; Braun et al., 2014, 2016), the illusion threshold to confirm a successful SoO and SoA induction across participants was set to ≥ 1.

**Table 2:** Questionnaire for assessment of subjective experiences.

Phenomenal target property	Statement
Sense of ownership	I felt as if the robotic hand was my own hand.
	I felt as if my real hand was at the position of the robotic hand.
	I felt as if the robotic hand was part of my body.
Sense of ownership (control questions)	I felt as if I no longer had a right hand; as if my right hand had disappeared.
	I felt as if I no longer had a left hand; as if my right hand had disappeared.
Sense of agency	I felt as if I was controlling the closing movements of the robotic hand.
	I felt as if I was controlling the opening movements of the robotic hand.
	I felt as if I could withhold any robotic hand movements.
Sense of agency (control question)	I felt as if the robotic hand was controlling my will.
Experiential realness	My imagined movements appeared as clear and detailed to my inner mind’s eye as if they actually happened.
	My imagined movements felt as vivid and real as if they actually happened.
	I forgot that I was just imagining and not actually executing the movements.
MI-action binding	I experienced my imagined hand extensions and the extensions of the robotic hand as inseparably linked with each other.
	I experienced my imagined hand flexions and the flexions of the robotic hand as inseparably linked with each other.
	I felt as if my imagined movements were happening at the position where the robotic hand was actually located.

*Note. Three statements addressed each phenomenal target property. In addition, control statements were included that included illusion-related statements but which did not capture the phenomenal experience of agency or ownership. Statements were read in counterbalanced to the participants, and the participants had to rate their level of agreement on a 7-point Likert scale*.

### Neurofeedback Performance Evaluation (Classification Accuracies)

#### Training Accuracies

Online training accuracies were calculated for all three classifiers with a five-fold block-wise cross-validation procedure. The calculation relied on the training block, using the same time segments as during classifier calibration. To statistically test whether the training accuracies were above chance level, a binomial statistic with *p* = 0.05 was used (Combrisson and Jerbi, 2015).

#### Feedback Accuracies

**Figure 3:**
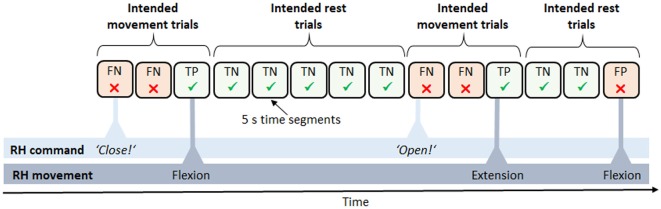
Derived trial structure in the neurofeedback block. Data were subdivided into trials of 5 s length between each pair of commands, e.g., “close” or “open.” Trials were defined as either intended movement trials, expecting a movement of the RH (e.g., a flexion), or intended rest trials, expecting the RH to maintain the current state. Accordingly, trials were classified as either success [true positive (TP) and true negative (TN)] or failure [false positive (FP) and false negative (FN)].

To evaluate performance during the feedback block, feedback accuracies were analyzed in each task. Additionally, the number of movements during rest phases was compared to the number of movements during move phases in the rest vs. move task. Since no fixed trial structure was given for the follow commands and announce commands task, we *post hoc* reconstructed a trial structure in order to calculate feedback accuracies (Figure 3). To this end, we segmented the data into 5 s intervals, relative to each command. We then defined two different trial types, intended movement trials and intended rest trials, and assigned each segment to one of the two types. A series of intended movement trials began as soon as a movement command was given by the experimenter or participant and lasted until the intended movement was finally carried out. A series of intended rest trials, in turn, started as soon as a movement was carried out and no further command was yet given. The intended movement trials in which a movement occurred were defined as true positive (TP) outcomes whereas those intended movement trials without any movement occurrence were defined as false negative (FN) outcomes. Likewise, the intended rest trials without any movement occurrences were defined as true negative (TN) outcomes whereas those intended rest trials with a movement occurrence were defined as false positive (FP) outcomes. To calculate overall feedback accuracies, the sum of all TP and TN trials was then divided by the total number of trials and multiplied by a hundred. The possible values thus ranged from zero, indicating that all trials failed, to 100, indicating only successful trials, that is, a perfect match of the participants’ intentions and the RH behavior. Regarding the feedback accuracies in the rest vs. move tasks, data were equally segmented into 5 s intervals, with move phases comprising only intended movement trials, and rest phases comprising only intended rest trials. To statistically test whether feedback accuracies in the follow commands and announce commands task were above chance level, an established statistical procedure relying on the classification accuracies” confidence intervals (CI) was used, with *p* = 0.05 (Billinger et al., 2012). Regarding the rest vs. move task, the same statistic as for the training accuracies was taken (Combrisson and Jerbi, 2015).

### Statistical Analyses

All statistical analyses were performed using bootstrapping procedures, as sample distributions, except for the ratings of the controls for SoA, ER and MIAB in the training block, and SoA and MIAB in the feedback block were non-normally distributed. Bootstrapping approaches have generally been shown to be a useful data analysis paradigm, particularly when assumptions underlying traditional statistical methods are violated, or when the sampling distributions of the test statistics are unknown. In these cases, an empirical sampling distribution for the statistic of interest is derived by repeatedly resampling (with replacement) from the sample at hand. In the context of null hypothesis significance testing, bootstrapping allows a data-driven approximate distribution of the test statistic, given the null hypothesis, to be obtained (instead of assuming a theoretical distribution; see e.g., Efron and Tibshirani, 1994, on using bootstrapping in null hypothesis significance testing).

To evaluate group and block differences in the subjective experiences, a two-way mixed ANOVA with bootstrapping, with the between-subject factor group (stroke patients vs. control participants) and within-subject factor block (training vs. feedback block), was run for each phenomenal target property (SoO, SoA, ER, MIAB).

To create the null condition, we first performed the centering of the within-subject factor. Next, we randomly resampled (with replacement) 18 cases of the centered data, and randomized the between-subjects factor. This yielded a bootstrapped data set, from which we computed the *F*-value of a two-way mixed ANOVA. We then repeated this process 3,000 times to create an empirical sampling distribution of *F*-values. Finally, the *F*-value of the original sample was placed within the corresponding empirical sampling F-distribution to determine the *p*-value. We report a significant result if the proportion of *F*-values larger than the observed one was below 5% (see e.g., Berkovits et al., 2000 on implementing bootstrapping in designs with repeated measures). All following two-way mixed ANOVA calculations were carried out using the same bootstrapping procedure.

Regarding the number of movements in the rest vs. move task, a two-way mixed ANOVA with bootstrapping, with the between-subjects factor group and the within-subjects factor phase (move phase vs. rest phase), was carried out. To evaluate group and task effects on feedback accuracies, a two-way ANOVA with bootstrapping, with the between-subjects factor group and within-subjects factor task (rest vs. move task, follow commands task, announce commands task), was conducted.

We conducted two-sided independent samples *t*-tests with bootstrapping to evaluate group differences in ERD latencies and CSP filter qualities. Two-sided independent samples *t-tests* with bootstrapping were also used as follow-up *t*-tests for significant effects resulting from the ANOVAs. To obtain bootstrapped samples for the *t*-tests, we pooled data of the stroke and control group, randomly generated two samples (with replacement), and estimated the *t-value* in a two-sided *t*-test. We repeated this procedure 3,000 times in order to estimate the t-distribution under the null hypothesis. We then calculated the probability of the *t-value* of our original data given this distribution and reported a significant result if it was below 5% (two-sided). Here, we report both *p-values* and 95% CI of the *t-value*.

Pearson correlation coefficients were calculated for pooled groups and blocks between all four phenomenal target properties. In addition, correlation coefficients were calculated for pooled groups between training accuracies and phenomenal target properties during the training block as well as between feedback accuracies and phenomenal target properties during the feedback block. All correlations were tested for significance again using bootstrapping. To create the null condition, we shuffled one variable while keeping the values of the other as in the original dataset. The subsequent steps were as described for the *t*-tests. All significance tests were Bonferroni-Holm-corrected for multiple comparisons. Statistical analyses were performed with Matlab 2017 and R 3.4.4 (R Core Team, 2018).

## Results

### Electrophysiological Results

To determine whether our classification algorithm operated on a physiologically-plausible EEG signal, and to check for ERD-related group differences, we conducted an offline TF analysis on the CSP-filtered EEG training block data as well as CSP filter quality check. More specifically, we assessed SMR power changes, ERD latencies and CSP filter qualities.

#### Power Changes in the SMR

**Figure 4:**
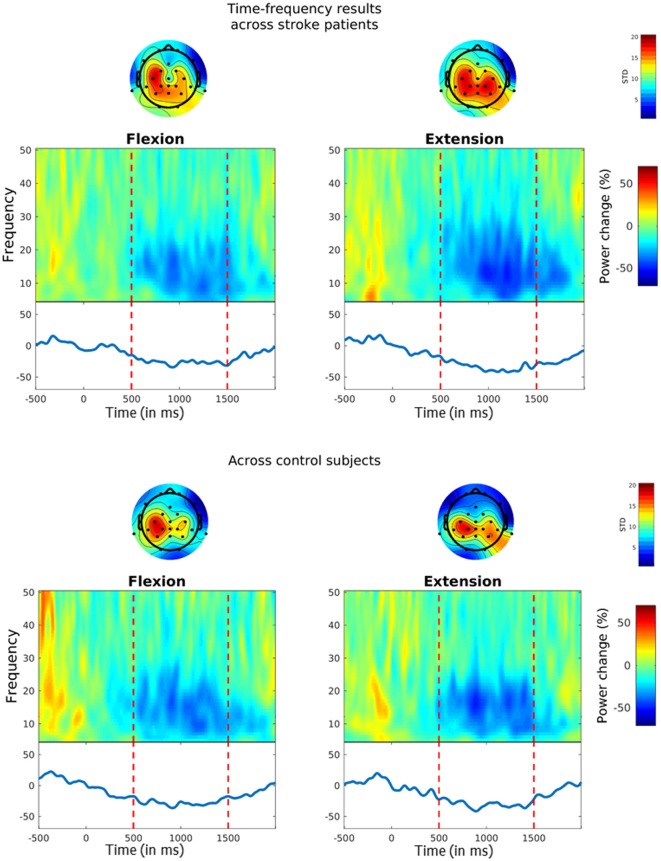
Event-related desynchronization (ERD) during MI. Time-frequency (TF) plots show the percentage change in power from baseline (i.e. from −0.5 s to 0 s) for MI flexion trials (left panels) and MI extension trials (right panels). MI started at time point zero and was performed for 1.5 s. Vertical lines indicate the chosen time interval for the statistical analysis (i.e. from 0.5 s to 1.5 s). The solid blue line on the bottom reflects MI-related power changes within the 10–25 Hz SMR frequency range. Topoplots above the TF plots show the standard deviations across the chosen common spatial pattern (CSP)-filters for each channel.

The TF plots across subjects for the chosen CSP channels are shown in Figure 4. For each plot, mean percent log power changes across trials are depicted for each frequency bin. As can be seen, both stroke patients (upper plots) and healthy controls (lower plots) showed a power reduction within the SMR’s 10–25 Hz frequency range during MI flexion trials (left plots) and MI extension trials (right plots). In the stroke patients, this power decrease amounted to 26.31% (*SD* = 19.94%) in the flexion trials and to 33.94% (*SD* = 20.01%) in the extension trials, whereas in the healthy participants’ SMR power decreased by 28.09% (*SD* = 24.58%) in the flexion trials and by 30.73% (*SD* = 14.97%) in the extension trials. Hence, the expected ERD pattern was clearly inducible for both groups and both MI types. To evaluate potential differences between groups or periods, a mixed two-way ANOVA with bootstrapping was conducted on the mean percent log power change between 10–25 Hz, which neither revealed a main effect of group, *F*_(1,16)_ = 0.01, *p* = 0.931, nor a main effect of period, *F*_(1,16)_ = 1.1, *p* = 0.327, and also no interaction, *F*_(1,16)_ = 0.26, *p* = 0.633.

#### ERD Latencies

ERD latencies were defined as the time interval between MI period onset and ERD onset, whereby an ERD onset was defined as the time-point at which the 10–25 Hz SMR power was reduced by at least 30%. Average ERD latencies across flexion and extension trials amounted to *M* = 631.58 ms (*SD* = 141.21) in the stroke patients and *M* = 506.56 ms (*SD* = 126.59) in the control participants. For evaluating potential group differences, a two-sided independent samples *t*-test with bootstrapping was conducted, which, revealed a non-significant trend for longer ERD latencies in stroke patients as compared to control participants, *t*_(16)_ = −1.98 [95% CI (−2.19, 2.17)], *p* = 0.069.

#### CSP Filter Plausibility

Based on our criteria for CSP filter evaluation (see Supplementary Material S1), we derived a CSP filter quality score for each participant, whereby values ≥10 were deemed to indicate a high CSP filter quality. Results reveal that three, stroke patients and four control participants reached a value of 10 or above, while on average, the CSP filter quality amounted to *M* = 8.63 (*SD* = 2.77) in the stroke patients and *M* = 8.22 (*SD* = 2.73) in the healthy participants. A two-sided independent samples *t*-test with bootstrapping revealed no differences in the quality of the selected CSP filters between stroke patients and control participants *t*_(16)_ = −0.3 [95% CI (−2.26, 2.06)], *p* = 0.761.

### Questionnaire Results

**Figure 5:**
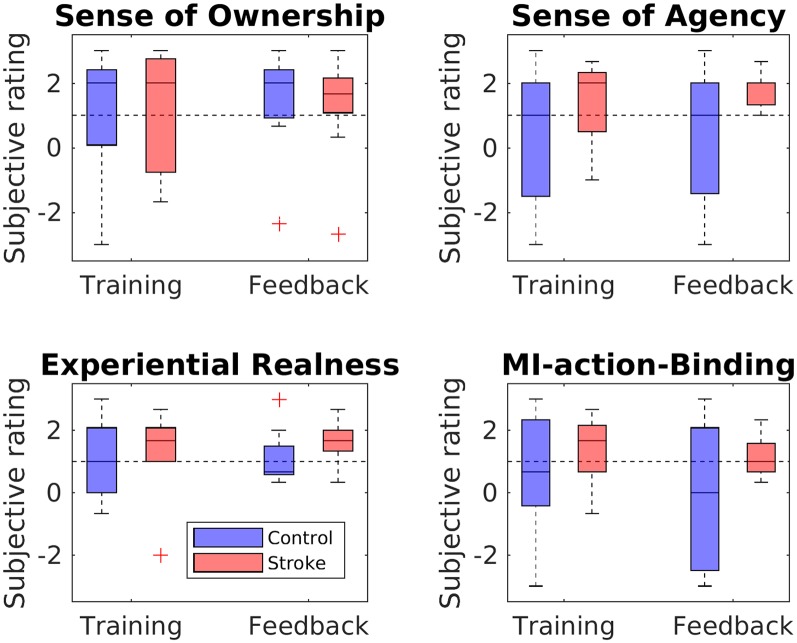
Questionnaire ratings for each phenomenal target property. Boxplots depict questionnaire ratings for the training and feedback blocks, separately for stroke patients and healthy controls. The black dashed line indicates successful RHI induction on group level.

The perceived SoO, SoA, ER, and MIAB levels are depicted in Figure 5. Each phenomenal target property was considered as successfully induced with an average value of ≥ 1 on a group level.

#### Sense of Ownership

The SoO-induction criterion was reached in both groups (patients and controls) and both blocks (training and feedback), while all SoO control questions showed values around zero or less, thus refuting any suggestion of response bias. The highest SoO level was reported by the stroke patients during the neurofeedback block (*M* = 1.26, *SD* = 1.66), whereas the lowest SoO level was reported by the healthy controls during the training phase (*M* = 1.00, *SD* = 1.62). A mixed two-way ANOVA with bootstrapping revealed no main effect of group, *F*_(1,16)_ = 0.00, *p* = 0.964, no main effect of phase, *F*_(1,16)_ = 0.81, *p* = 0.402, and no interaction, *F*_(1,16)_ = 0.32, *p* = 0.595.

#### Sense of Agency

The SoA-induction criterion was met for the stroke group in the training phase (*M* = 1.33; *SD* = 1.29) and feedback phase (*M* = 1.59; *SD* = 0.52). By contrast, for the control group, the criterion was not reached either in the training (*M* = 0.33; *SD* = 2.2) or in the feedback (*M* = 0.37; *SD* = 2.0) phase. Mean values of the SoA control items were around zero in both groups (stroke group: *M* = 0.28, *SD* = 1.56; control group: *M* = −0.83, *SD* = 1.8), thereby refuting any suggestion of response bias. The mixed two-way ANOVA with bootstrapping revealed neither a main group effect, *F*_(1,16)_ = 2.41, *p* = 0.157, nor a main effect of phase, *F*_(1,16)_ = 0.25, *p* = 0.647, nor an interaction between group and phase, *F*_(1,16)_ = 0.14, *p* = 0.718.

#### Experiential Realness

The ER-induction criterion was met for both groups in both the training phase (stroke group: *M* = 1.33, *SD* = 1.37; control group: *M* = 1.15, *SD* = 1.25) and the feedback phase (stroke group: *M* = 1.63, *SD* = 0.66; control group: *M* = 1.11, *SD* = 0.88). A two-way mixed ANOVA with bootstrapping revealed neither a main effect of group, *F*_(1,16)_ = 0.58, *p* = 0.473, nor of phase, *F*_(1,16)_ = 0.38, *p* = 0.552, nor an interaction, *F*_(1,16)_ = 0.62, *p* = 0.463.

#### MI-Action Binding

Whereas the patient group met the MIAB-induction criterion in both phases (training: *M* = 1.41, *SD* = 1.2; feedback: *M* = 1.19, *SD* = 0.71), the control group did so only in the feedback phase (training: *M* = 0.6; *SD* = 1.89; feedback: *M* = −0.07; *SD* = 2.3). A mixed two-way ANOVA with bootstrapping revealed neither a main effect of group, *F*_(1,16)_ = 2.46, *p* = 0.153, nor of phase, *F*_(1,16)_ = 1.2, *p* = 0.327, nor an interaction, *F*_(1,16)_ = 0.3, *p* = 0.602.

### Performance Results

#### Training Accuracies

**Figure 6:**
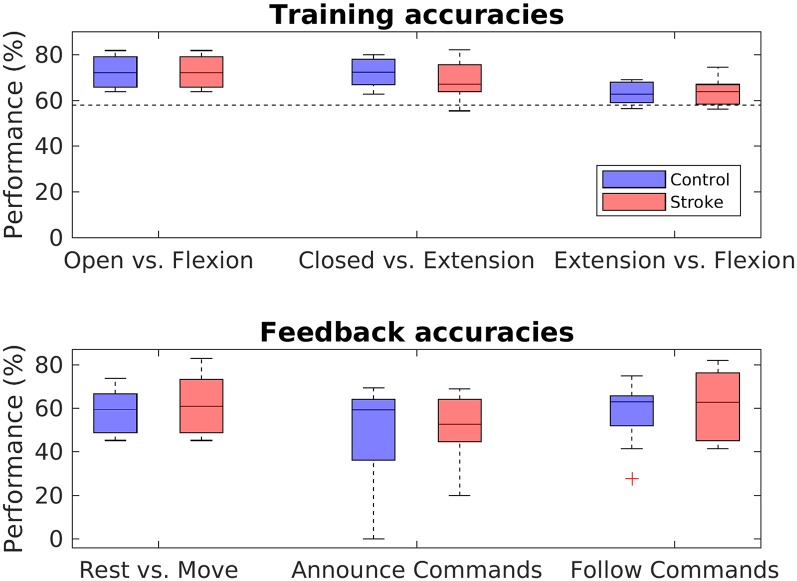
Performance analysis. Boxplots depict the crossfold-validation-based training accuracies of the three classifiers from the training phase (upper panel), and the feedback accuracies for the three different MI-tasks of the neurofeedback phase (lower panel). The black dashed line in the upper panel indicates the statistical chance level (*p* = 0.05).

Training accuracies were calculated for all three classifiers with a five-fold block-wise cross-validation procedure using the EEG data from the training block. Accuracies for both groups and all three classifiers are shown in the upper panel of Figure 6. In the stroke group, overall training accuracies were significantly above chance level (*α* = 0.05, Combrisson and Jerbi, 2015) in 24 of the 27 derived accuracies (three classifiers times nine stroke patients), giving a proportion of 88.89%. In the control group, 25 of the 27 derived accuracies were above chance-level, corresponding to a proportion of 92.59%. In order to evaluate differences in groups and classifiers, a mixed two-way ANOVA with bootstrapping with the within-subject factor classifier and the between-subject factor group was conducted, revealing a main effect for the classifiers, *F*_(1,16)_ = 12.98, *p* = 0.003, but no main effect for group, *F*_(1,16)_ = 1.24, *p* = 0.285. *Post hoc* two-sided independent-samples *t*-tests with bootstrapping revealed a difference between the training accuracies of the opened vs. flexion classifier and the extension vs. flexion classifier, *t*_(16)_ = −3.26 [95% CI (−2.07, 2.01)], *p* = 0.003, *d* = 0.77, as well as between the closed vs. extension classifier and extension vs. flexion classifier, *t*_(16)_ = 3.22 [95% CI (−2.01, 2.02)], *p* = 0.005, *d* = 0.76 (all values passing the Bonferroni adjustment of alpha = 0.025).

#### Feedback Accuracies

In order to assess the performance during the feedback block, feedback accuracies were analyzed for each task. Feedback accuracies are depicted for both groups and all three tasks in the lower panel of Figure 6. The average feedback accuracy across tasks was *M* = 57.95% (*SD* = 12.74) in stroke patients and *M* = 54.31% (*SD* = 15.16) in control participants. Differentiating between the tasks, feedback accuracies varied highly among participants in both groups, with values from 20% to 82,93% in stroke patients and from 0% to 75% in control participants. In both the control and the patient group, accuracies were significantly above chance level in 18 of the 27 derived accuracies, giving a portion of 66.67%. In order to evaluate differences between groups and tasks, a two-way mixed ANOVA with bootstrapping was conducted, revealing no main effect of task, *F*_(1,16)_ = 5.13, *p* = 0.075, no main effect for group, *F*_(2,15)_ = 0.32, *p* = 0.587, and no interaction effect between group and task, *F*_(3,15)_ < 0.001, *p* = 0.978.

#### Robotic Hand Movements in Rest vs. Move Task

**Figure 7:**
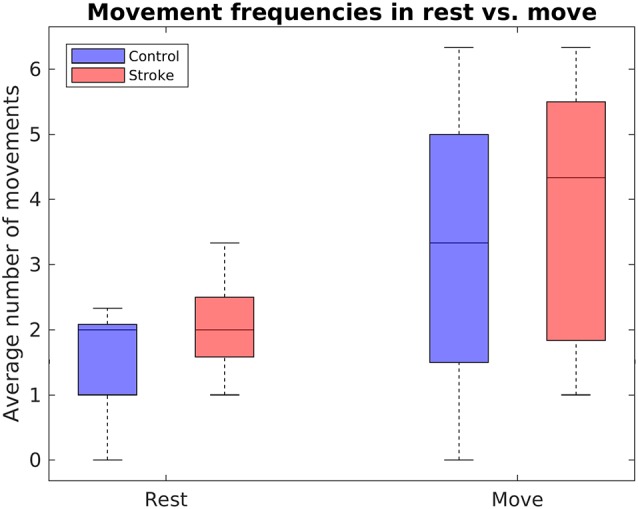
Boxplots depict the mean number of RH movements within the rest vs. move task, separately for stroke patients and healthy controls.

In addition to evaluating the feedback accuracies, performance has been further assessed by comparing the number of movements during rest phases to the number of movements during move phases in the rest vs. move task. Figure 7 displays the mean number of movements during rest and movement phases of the rest vs. move task. A mixed two-way ANOVA with bootstrapping revealed an effect of phase (*F*_(1,16)_ = 13.84, *p* = 0.003), in that participants conducted fewer movements during the rest phases (*M* = 1.76, *SD* = 0.86) than movement phases (*M* = 3.50, *SD* = 2.12). In contrast, no main effect of group, *F*_(1,16)_ = 1.52, *p* = 0.252, nor any interaction effect were found, *F*_(1,16)_ = 0.16, *p* = 0.697.

### Correlation Analysis

In order to assess the relationships between our various experimental variables, Pearson correlation coefficients were calculated for pooled groups and blocks between, first, all four phenomenal target properties, second, training accuracies and phenomenal target properties during the training block, and third, feedback accuracies and phenomenal target properties during the feedback block. Significant positive correlations across groups and blocks were found between all four phenomenological constructs, all of which depict large effect-sizes (see Table 3). Apart from that, no other significant correlations were found.

**Table 3:** Significant correlations between phenomenal target properties.

	*r*	*R*^2^	*t*_(16)_	95% CI	*p*
SoO vs. SoA	0.56	31%	4.92	(−1.84, 2.18)	<0.001
SoO vs. ER	0.76	58%	6.91	(−1.89, 2.11)	<0.001
SoO vs. MIAB	0.62	38%	4.57	(−1.90, 2.20)	<0.001
SoA vs. ER	0.71	50%	5.87	(−1.91, 2.13)	<0.001
SoA vs. MIAB	0.82	67%	8.31	(−1.88, 2.16)	<0.001
ER vs. MIAB	0.73	53%	6.24	(−1.87, 2.24)	<0.001

*Note. Table includes the Pearson correlation coefficients (r), the R-squared, the t-value (t) with 16^°^ of freedom, the 95% confidence interval (95% CI) of the t-value, and the p-value (p)*.

## Discussion

The aim of the present study was to investigate the feasibility of a self-paced and embodiable NF-MIT. To this end, an anthropomorphic RH was integrated into an EEG-based BCI and used for neurofeedback provision. Within three different neurofeedback tasks, stroke patients and healthy controls freely attempted to control the RH’s movement behavior in a self-paced manner, using MI.

### General Feasibility

The general feasibility of our NF-MIT was investigated on the electrophysiological, phenomenological, and performance level. On the electrophysiological level, across trials the expected ERD pattern of a right-handed MI task was evident in most of our participants. That is, for most of our participants, the TF analysis revealed a typical MI-related decrease in 8–30 Hz oscillatory brain activity over the sensorimotor areas. Results from the CSP filter analysis were more mixed: While only 38% of our participants reached our threshold for full CSP-filter plausibility (≥10), most remaining participants were close to our (rather restrictive) cut-off value (see “Discussion” section below). Moreover, the criteria for CSP-filter plausibility pertain to the discriminability between compared mental states rather than to whether or not the filters are anatomically plausible in the first place. In other words: while our chosen CSP-filters were rather poor in discriminating between the different mental states, their spectrotemporal filter characteristics were as expected. Taking these findings together, we thus conclude that the classification algorithm operated on noisy, but electrophysiologically-plausible, MI-related brain signals and not merely on some technical artifacts.

Regarding phenomenology, our questionnaire results revealed that the induction criterion was reached on the group level in both blocks and both groups for the target properties SoO and ER. With respect to MIAB, the illusion criterion was met in both groups during the training block, but only in the stroke group during the feedback block. Regarding SoA, the illusion criterion was met during both blocks for the stroke patients, but in neither block for the healthy controls (see “Discussion” section below). These findings demonstrate that the RH could be embodied in the participants’ phenomenal body scheme to a reasonable degree. That is, most participants of both groups experienced the RH as part of their own body and their MI acts as close to real. Moreover, the majority experienced the MI act as perceptually fused with the RH motion percept. Regarding the subjective ratings derived from the training block, these results replicate our former study (Braun et al., 2016). Here, we similarly showed that within a fixed trial structure, illusory SoO and SoA, as well as ER and MIAB, may be achieved over an external device by the approximate synchrony of imagined limb movements and observed RH movements. As pertains to the subjective ratings derived from the neurofeedback block, we find a similarly successful induction of the four phenomenal target properties, but this time in a scenario without a fixed trial structure. This shows that even in the presence of significant temporal mismatches between the imagined limb movements and RH motion behavior, the RH was still phenomenally embodiable. This, in turn, suggests that either the proportion of mismatches is small enough for SoO, SoA, ER and MIAB not to be disrupted, or these four phenomenal constructs are sufficiently robust to violations of expected RH actions.

Regarding the performance level, we investigated classification accuracies from both the training block (training accuracies) and feedback block (feedback accuracies). For the training block, most of our participants, regardless of whether they belonged to the control or stroke group, achieved training accuracies that exceeded the statistical chance level. This finding is in line with the typical MI classification outcomes of traditional NF-MIT paradigms (see e.g., Yong and Menon, 2015) and also with the findings of our former aRHI study (Braun et al., 2016). The finding indicates that the MI-induced ERD pattern was not only evident across trials as seen in the ERD findings, but was reliable enough to be also detectable at the single-trial level. Moreover, the finding shows that our classification algorithm was not only suitable for healthy controls but worked equally well with stroke patients. The difference in classifier training accuracy between extension vs. flexion and the other two classifiers is probably because it is harder to distinguish two types of movement in EEG than it is to distinguish movement from non-movement.

For the feedback block, we found that most participants were able to achieve at least some level of control over the RH’s actions. That is, although mean feedback accuracies across tasks were rather poor (~58% in stroke patients, ~54% in healthy controls), almost all participants (nine of nine stroke patients and eight of nine healthy controls) performed at least one neurofeedback task above chance level. More generally, the majority of all accuracies (~67% in both groups) obtained in the three tasks—rest vs. move, announce commands and follow commands—exceeded that threshold. Furthermore, in the rest vs. move task, both groups showed significantly fewer RH movements in the rest phases than in the movement phases. Taking these findings together, this shows that, in principle, it is possible to use a self-paced paradigm for RH control. To our knowledge, this is the first study to have demonstrated this.

It should be pointed out that we cannot claim any superiority of an embodiable over an abstract feedback signal under self-paced NF-MIT. This was not, however, the principal research objective in the present study. Rather, we intended to investigate whether an EFS is, in principle, feasible in a self-paced neurofeedback paradigm. Future studies are necessary to compare abstract and EFSs, and to investigate under which circumstances the different feedback types are more suitable.

### Group Differences

We found differences between stroke patients and control participants on the electrophysiological and phenomenological levels, but not on the performance level.

On the electrophysiological level, we found a non-significant trend for a delayed ERD onset in stroke patients as compared to healthy controls. While distinct lateralized ERD patterns in control participants as compared to stroke patients have been widely addressed in previous studies (Feydy et al., 2002; Scherer et al., 2007; Braun et al., 2017), few did so with respect to distinct ERD latencies in the context of NF-MIT. This might be due to highly varying study designs and a few direct comparisons of stroke patients with matched control participants. Schaechter (2004), for instance, reported a prolonged latency of motor-evoked potentials after the infarct. Moreover, findings by Crone et al. (1998) showed a longer ERD latency for ipsilateral activation patterns as compared to contralateral activation. It has been suggested that delays in ERD onset result from slower information processing in damaged brain structures (Leocani and Comi, 2006). Although we remain cautious in interpreting the trend we found here for a delayed ERD onset, it is thus, in principle, compatible with the existing evidence.

As regards the phenomenological level, we found a difference between groups for SoA. That is, whereas the illusion criterion was met during both blocks for the stroke patients, it was not met in either block for the healthy controls. A speculative explanation might be that stroke patients generally have a vaguer percept of the affected hand, leading them to more quickly experience SoA. As functional reliability in neural tissues may be damaged in stroke patients, these patients may be accustomed to spending more effort to control their own affected hand.

### Correlation Analysis

In line with our former study (Braun et al., 2016), we observed high correlations between SoO, SoA, ER and MIAB. This suggests that these subjective measures do not relate to separate, but rather to overlapping and interacting aspects of phenomenal experience (for a discussion, see Braun et al., 2016). However, we did not find significant correlations between any subjective measure and the classification accuracies. Contradicting our argumentation for an EFS, a direct relationship between the participants’ perceived level of RH embodiment and neurofeedback performances can thus not be demonstrated on a statistical level. Given the low sample sizes, these null findings may, however, be due to a lack of statistical power or due to a suboptimal EFS implementation (see “Study Limitations” section). Taking into account existing evidence supporting the beneficial effects of an EFS (see “Introduction” section), we believe that the lack of correlation does not render EFS useless, as we argue in the following.

#### Arguments for an Embodiable Feedback Signal

One benefit of an EFS could be that it facilitates causal inference (Shams and Beierholm, 2010). If the provided neurofeedback signal closely enough resembles the MI act performed, in both time and space, the MI percept and the visual percept induced by the neurofeedback signal could possibly be better fused. Consequently, the brain could infer a common cause for both percepts.

A second advantage could be that the perceptual fusion just described opens up the possibility for inducing SoO, which, in turn, might provoke SoA (Kalckert and Ehrsson, 2012, 2014; Braun et al., 2014, 2016). As has been repeatedly demonstrated (Perez-Marcos et al., 2009; Sanchez-Vives et al., 2010; Braun et al., 2016), SoO is inducible by merely imagining limb movements in approximate temporal synchrony to observed hand movements. A neurofeedback signal that is inherently linked to the BCI-user’s own body and its voluntary movements should increase the compliance with the NF-MIT and help to convince the BCI-user about the effectiveness of the training, thereby improving his or her motivation.

A third benefit might be that an EFS potentially reduces the patient’s cognitive workload since the cognitively-demanding task of mentally rehearsing limb movements could be bottom-up facilitated by the EFS (as with mirror visual feedback; Ramachandran and Altschuler, 2009). To be clear, for healthy participants such cognitive offloading might be unnecessary or perhaps even a hindrance. The major target group of NF-MIT is, however, stroke patients whose MI abilities are often impaired, and here much more uncertainty exists as to what these BCI-users actually imagine.

Fourth, an EFS may require less abstract thinking by the BCI-user than an abstract neurofeedback signal. Whereas with an abstract neurofeedback signal the BCI-user has to understand that the signal shown on the computer screen relates to his or her own MI-act, this act of abstraction would not be needed with an EFS.

Many arguments may thus be found in favor of an embodiable rather than an abstract neurofeedback signal. We acknowledge, however, that these arguments assume an idealized scenario, in which the EFS sufficiently matches the mental acts performed. In what way such an EFS can be realized in practice, awaits further empirical validation.

### Study Limitations

Our phenomenological results support the hypothesis that the RH as a feedback signal was embodiable. Yet, an RH adjusted to user-specific sizes might have improved the embodiment—we recruited all genders, but used a larger RH for all participants, originally modeled from a male hand.

Regarding the rather poor performance accuracies in the neurofeedback block, several reasons might account for the deviation between intended and observed BCI output. First, our participants could just have had rather poor MI abilities. That is, they were not sufficiently able to vividly imagine the required limb movements, and as a consequence, were over-challenged with the given MI task. For the present study, we, cannot follow up on this possibility, given that we did not include an MI ability or severity questionnaire. It should, however, be noted that former studies found no (Rimbert et al., 2019) or rather moderate (Vuckovic and Osuagwu, 2013; Marchesotti et al., 2016) relations between subjective MI ability measures and BCI literacy.

Second, the rather poor neurofeedback performances might have been caused by shortcomings of the participants’ ability to modulate their SMRs. Here, an indirect measure of SMR control is given by the derived brain activity patterns and ERD analysis. As expected, MI-related activation patterns over sensorimotor areas could be observed in both groups, with moderate and mostly above-chance level mean training accuracies across classifiers.

Third, there may have been deficits in the interpretation and translation of brain activity into control signals (Mason et al., 2006). Our classification algorithm was based on the derivation of CSP filters. We found electrophysiologically plausible, yet highly varying, filters in both groups. In order to achieve a better classification, enhanced reliability for obtaining high-quality CSPs is required (Ang et al., 2008). Overall, we consider advanced machine learning algorithms a key for future research in NF-MIT and, ultimately, for advancing NF-MIT clinical application. Without substantial improvements in EEG-based SMR signal extraction, this may remain a challenging goal.

Fourth, it should be noted that during the training block, the RH always moved in synchrony with the imagination of the participant’s own limb movement. From a phenomenological perspective, we consider this design aspect appropriate, in order to bottom-up facilitate the patient’s MI process and for keeping the training and neurofeedback block as similar as possible. From a signal processing perspective, however, this design aspect has a caveat, in that limb movement observation and limb movement imagination may both induce an ERD. As a consequence, it remains unknown as to how far the ERD pattern observed is driven by the limb movement imagination, and not exclusively by the limb movement observation. If only the latter were the case, this would cast problems on the BCI, since it requires a brain signal that can be mentally self-induced, independent from external sensory stimulation. For the present experiment, the above-chance level feedback accuracies during the NF block, however, indicate that the observed ERDs were at least partly driven by limb movement imaginations.

The method we adopted for CSP filter quality assessment was rather unstandardized. While we consider our six suggested CSP filter quality criteria valid, we are aware that many other criteria could be used instead, or in addition. Likewise, we are aware that our defined threshold of 10 is rather arbitrarily set and that other cut-off values could be defined. Despite these limitations, we nevertheless, consider our CSP-filter assessment procedure as an important step towards a physiologically-plausible CSP filter selection, given that most existing CSP-based NF-MIT paradigms just select CSP filters based on class-discriminability.

Interestingly, performance accuracies greatly varied, not only among participants but similar between the different neurofeedback tasks. This might be due to a lack of experience in NF-MIT, and therefore training phases prior to the actual NF-MIT might be beneficial to reduce exercise-dependent intraindividual variability in performance. Differences between subjects, on the other hand, suggest that the same NF-MIT paradigm may not be suitable for all participants, and prior training phases might be needed to assess an individuals’ potential for NF-MIT. Taking into account the participant’s suitability for NF-MIT based on a training paradigm might be another possibility for achieving less variability and superior feedback accuracies (de Vries and Mulder, 2007; Ono et al., 2013). Yet, it would also limit the generalizability of the application.

One potential drawback in our experimental design concerns differences in the task instructions between our training and feedback blocks. While in the training block, participants were instructed to just relax during the rest trials, in the feedback block, they were explicitly asked to employ mental strategies, such as counting numbers. Hence, with respect to phenomenal content, the resting states during the training block and feedback block differed, which might have reduced the classification algorithm’s reliability. The reason for giving different task instructions for the training and feedback resting periods was that, for the fixed and timely regular trial structure during the training block, we considered the refraining from MI to be much easier than during the self-paced feedback phase, when the rest periods were not of a fixed 5-s duration, but rather were of arbitrary length. In addition, we wanted to provide our participants with some cognitive strategies for potential benefit to their neurofeedback performances.

Finally, it should be recalled that MI instructions differed between the first (unreported here) and the second NF-MIT session. In spite of this difference in the MI tasks, general training effects might have occurred in the second session due to the first, as participants were more familiar with MI as well as the neurofeedback procedure. However, training effects of session one on session two do not in principle limit the feasibility of the second session, provided that all participants underwent both NF-MIT sessions in the same order, which was the case here.

Despite these drawbacks, we conclude that this study successfully demonstrates that healthy participants and stroke patients embodied an RH into their body scheme, and were able, to some modest degree, to control the RH in a self-paced setting. We hope this will motivate further research exploring the idea that an embodiable self-paced NF-MIT is beneficial in stroke motor rehabilitation.

## Data Availability Statement

The raw data supporting the conclusions of this article will be made available by the authors, without undue reservation, to any qualified researcher. Scripts used for the analysis are provided at https://github.com/nadinespy/SelfPacedEmbodiableNeurofeedback.

## Ethics Statement

This study was carried out in accordance with the recommendations of the University of Oldenburg ethics committee with written informed consent from all subjects.

## Author Contributions

NS and NB designed the experiment under the supervision of SD and JT. EB collected the data. NS and EB analyzed the data under the supervision of NB and SD. NS and NB wrote major parts of the manuscript. SD, HM, AP, EB, and JT contributed to, reviewed, and edited the manuscript.

## Conflict of Interest

The authors declare that the research was conducted in the absence of any commercial or financial relationships that could be construed as a potential conflict of interest.

## Footnotes

^1^https://github.com/sccn/labstreaminglayer

## References

[B1] Alimardani M. Nishio S. Ishiguro H. (2013). Humanlike robot hands controlled by brain activity arouse illusion of ownership in operators. Sci. Rep. 3:2396. 10.1038/srep02396 23928891 PMC3738936

[B100] Alimardani M. Nishio S. Ishiguro H. (2014). Effect of biased feedback on motor imagery learning in BCI-teleoperation system. Front. syst. Neurosci. 8:52 10.3389/fnsys.2014.00052 24782721 PMC3988391

[B2] Ang K. K. Chin Z. Y. Zhang H. Guan C. (2008). “Filter bank common spatial pattern (FBCSP),” in Proceedings of the 2008 IEEE International Joint Conference on Neural Networks (IEEE World Congress on Computational Intelligence) (Hong Kong, China: IEEE), 2390–2397.

[B3] Bell A. J. Sejnowski T. J. (1995). An information-maximization approach to blind separation and blind deconvolution. Neural Comput. 7, 1129–1159. 10.1162/neco.1995.7.6.1129 7584893

[B4] Berkovits I. Hancock G. R. Nevitt J. (2000). Bootstrap resampling approaches for repeated measure designs: relative robustness to sphericity and normality violations. Educ. Psychol. Meas. 60, 877–892. 10.1177/00131640021970961

[B5] Billinger M. Daly I. Kaiser V. Jin J. Allison B. Z. Müller-Putz G. R. (2012). “Is it significant? Guidelines for reporting BCI performance,” in Towards Practical Brain-Computer Interfaces, eds Allison B. Z. Dunne S. Leeb R. Millan J. Nijholt A. (Berlin, Heidelberg: Springer), 333–354.

[B6] Blankertz B. Tomioka R. Lemm S. Kawanabe M. Müller K.-R. (2008). Optimizing spatial filters for robust single-trial analysis. IEEE Signal Process. Mag. 25, 41–56. 10.1109/msp.2008.4408441

[B7] Braun N. Emkes R. Thorne J. D. Debener S. (2016). Embodied neurofeedback with an anthropomorphic robotic hand. Sci. Rep. 6:37696. 10.1038/srep37696 27869190 PMC5116625

[B8] Braun N. Kranczioch C. Liepert J. Dettmers C. Zich C. Büsching I.. (2017). Motor imagery impairment in postacute stroke patients. Neural Plast. 2017:4653256. 10.1155/2017/4653256 28458926 PMC5387846

[B9] Braun N. Thorne J. D. Hildebrandt H. Debener S. (2014). Interplay of agency and ownership: the intentional binding and rubber hand illusion paradigm combined. PLoS One 9:e111967. 10.1371/journal.pone.0111967 25369067 PMC4219820

[B10] Cervera M. A. Soekadar S. R. Ushiba J. Millán J. D. R. Liu M. Birbaumer N.. (2018). Brain-computer interfaces for post-stroke motor rehabilitation: a meta-analysis. Ann. Clin. Transl. Neurol. 5, 651–663. 10.1002/acn3.544 29761128 PMC5945970

[B11] Combrisson E. Jerbi K. (2015). Exceeding chance level by chance: the caveat of theoretical chance levels in brain signal classification and statistical assessment of decoding accuracy. J. Neurosci. Methods 250, 126–136. 10.1016/j.jneumeth.2015.01.010 25596422

[B12] Crone N. E. Miglioretti D. L. Gordon B. Sieracki J. M. Wilson M. T. Uematsu S.. (1998). Functional mapping of human sensorimotor cortex with electrocorticographic spectral analysis. I. α and β event-related desynchronization. Brain 121, 2271–2299. 10.1093/brain/121.12.2271 9874480

[B13] de Vries S. Mulder T. (2007). Motor imagery and stroke rehabilitation: a critical discussion. J. Rehabil. Med. 39, 5–13. 10.2340/16501977-0020 17225031

[B14] Debener S. Ullsperger M. Siegel M. Fiehler K. Von Cramon D. Y. Engel A. K. (2005). Trial-by-trial coupling of concurrent electroencephalogram and functional magnetic resonance imaging identifies the dynamics of performance monitoring. J. Neurosci. 25, 11730–11737. 10.1523/JNEUROSCI.3286-05.2005 16354931 PMC6726024

[B15] Delorme A. Makeig S. (2004). EEGLAB: an open source toolbox for analysis of single-trial EEG dynamics including independent component analysis. J. Neurosci. Methods 134, 9–21. 10.1016/j.jneumeth.2003.10.009 15102499

[B16] Delorme A. Sejnowski T. Makeig S. (2007). Enhanced detection of artifacts in EEG data using higher-order statistics and independent component analysis. NeuroImage 34, 1443–1449. 10.1016/j.neuroimage.2006.11.004 17188898 PMC2895624

[B101] Dreiseitl S. Ohno-Machado L. (2002). Logistic regression and artificial neural network classification models: a methodology review. J. Biomed. Inform. 35, 352–359. 10.1016/S1532-0464(03)00034-0 12968784

[B17] Efron B. Tibshirani R. J. (1994). An Introduction to the Bootstrap. Chapman & Hall: CRC Press, New York.

[B18] Feydy A. Carlier R. Roby-Brami A. Bussel B. Cazalis F. Pierot L.. (2002). Longitudinal study of motor recovery after stroke: recruitment and focusing of brain activation. Stroke 33, 1610–1617. 10.1161/01.str.0000017100.68294.52 12053000

[B19] Foglia L. Wilson R. A. (2013). Embodied cognition. Wiley Interdiscip. Rev. Cogn. Sci. 4, 319–325. 10.1002/wcs.1226 26304209

[B20] Grefkes C. Ward N. S. (2014). Cortical reorganization after stroke: how much and how functional? Neuroscientist 20, 56–70. 10.1177/1073858413491147 23774218

[B21] Kalckert A. Ehrsson H. H. (2012). Moving a rubber hand that feels like your own: a dissociation of ownership and agency. Front. Hum. Neurosci. 6:40. 10.3389/fnhum.2012.00040 22435056 PMC3303087

[B22] Kalckert A. Ehrsson H. H. (2014). The moving rubber hand illusion revisited: comparing movements and visuotactile stimulation to induce illusory ownership. Conscious. Cogn. 26, 117–132. 10.1016/j.concog.2014.02.003 24705182

[B23] Kilteni K. Normand J. M. Sanchez-Vives M. V. Slater M. (2012). Extending body space in immersive virtual reality: a very long arm illusion. PLoS One 7:e40867. 10.1371/journal.pone.0040867 22829891 PMC3400672

[B24] Kothe C. A. Makeig S. (2013). BCILAB: a platform for brain-computer interface development. J. Neural Eng. 10:056014. 10.1088/1741-2560/10/5/056014 23985960

[B25] Leocani L. Comi G. (2006). Movement-related event-related desynchronization in neuropsychiatric disorders. Prog. Brain Res. 159, 351–366. 10.1016/s0079-6123(06)59023-5 17071242

[B26] Lincoln N. B. Jackson J. M. Adams S. A. (1998). Reliability and revision of the nottingham sensory assessment for stroke patients. Physiotherapy 84, 358–365. 10.1016/s0031-9406(05)61454-x

[B27] Lotte F. Larrue F. Mühl C. (2013). Flaws in current human training protocols for spontaneous brain-computer interfaces: lessons learned from instructional design. Front. Hum. Neurosci. 7:568. 10.3389/fnhum.2013.00568 24062669 PMC3775130

[B28] Ma K. Hommel B. (2013). The virtual-hand illusion: effects of impact and threat on perceived ownership and affective resonance. Front. Psychol. 4:604. 10.3389/fpsyg.2013.00604 24046762 PMC3764400

[B29] Ma K. Lippelt D. P. Hommel B. (2017). Creating virtual-hand and virtual-face illusions to investigate self-representation. J. Vis. Exp. 121:e54784. 10.3791/54784 28287602 PMC5407672

[B30] Marchesotti S. Bassolino M. Serino A. Bleuler H. Blanke O. (2016). Quantifying the role of motor imagery in brain-machine interfaces. Sci. Rep. 6:24076. 10.1038/srep24076 27052520 PMC4823701

[B31] Mason S. Kronegg J. Huggins J. Fatourechi M. Schlögl A. (2006). Evaluating the performance of self-paced brain-computer interface technology. Tech Rep. (Vancouver, BC, Canada: Neil Squire Soc.), 1–60.

[B32] Nasreddine Z. S. Phillips N. A. Bedirian V. Charbonneau S. Whitehead V. Collin I.. (2005). The montreal cognitive assessment, MoCA: a brief screening tool for mild cognitive impairment. J. Am. Geriatr. Soc. 53, 695–699. 10.1111/j.1532-5415.2005.53221.x 15817019

[B33] Ono T. Kimura A. Ushiba J. (2013). Daily training with realistic visual feedback improves reproducibility of event-related desynchronisation following hand motor imagery. Clin. Neurophysiol. 124, 1779–1786. 10.1016/j.clinph.2013.03.006 23643578

[B34] Perez-Marcos D. Slater M. Sanchez-Vives M. V. (2009). Inducing a virtual hand ownership illusion through a brain-computer interface. Neuroreport 20, 589–594. 10.1097/wnr.0b013e32832a0a2a 19938302

[B35] Pichiorri F. Morone G. Petti M. Toppi J. Pisotta I. Molinari M.. (2015). Brain-computer interface boosts motor imagery practice during stroke recovery. Ann. Neurol. 77, 851–865. 10.1002/ana.24390 25712802

[B36] Ramachandran V. S. Altschuler E. L. (2009). The use of visual feedback, in particular mirror visual feedback, in restoring brain function. Brain 132, 1693–1710. 10.1093/brain/awp135 19506071

[B37] Ramoser H. Müller-Gerking J. Pfurtscheller G. (2000). Optimal spatial filtering of single-trial EEG during imagined hand movement. IEEE Trans. Rehabil. Eng. 8, 441–446. 10.1109/86.895946 11204034

[B102] R Core Team (2018). R: A Language and Environment for Statistical Computing. Vienna, Austria: R Foundation for Statistical Computing Available online at: http://www.R-project.org/.

[B38] Rimbert S. Gayraud N. Bougrain L. Clerc M. Fleck S. (2019). Can a subjective questionnaire be used as brain-computer interface performance predictor? Front. Hum. Neurosci. 12:529. 10.3389/fnhum.2018.00529 30728772 PMC6352609

[B39] Sanchez-Vives M. V. Spanlang B. Frisoli A. Bergamasco M. Slater M. (2010). Virtual hand illusion induced by visuomotor correlations. PLoS One 5:e10381. 10.1371/journal.pone.0010381 20454463 PMC2861624

[B40] Sanford J. Moreland J. Swanson L. R. Stratford P. W. Gowland C. (1993). Reliability of the Fugl-Meyer assessment for testing motor performance in patients following stroke. Phys. Ther. 73, 447–454. 10.1093/ptj/73.7.447 8316578

[B41] Schaechter J. D. (2004). Motor rehabilitation and brain plasticity after hemiparetic stroke. Prog. Neurobiol. 73, 61–72. 10.1016/j.pneurobio.2004.04.001 15193779

[B42] Scherer R. Lee F. Schlogl A. Leeb R. Bischof H. Pfurtscheller G. (2008). Toward self-paced brain-computer communication: navigation through virtual worlds. IEEE Trans. Biomed. Eng. 55, 675–682. 10.1109/tbme.2007.903709 18270004

[B43] Scherer R. Mohapp A. Grieshofer P. Pfurtscheller G. Neuper C. Scherer R. (2007). Sensorimotor EEG patterns during motor imagery in hemiparetic stroke patients. Int. J. Bioelectromagn. 9, 155–162.

[B44] Shams L. Beierholm U. R. (2010). Causal inference in perception. Trends Cogn. Sci. 14, 425–432. 10.1016/j.tics.2010.07.001 20705502

[B45] Sitaram R. Ros T. Stoeckel L. Haller S. Scharnowski F. Lewis-Peacock J.. (2017). Closed-loop brain training: the science of neurofeedback. Nat. Rev. Neurosci. 18, 86–100. 10.1038/nrn.2016.164 28003656

[B46] Slater M. Perez-Marcos D. Ehrsson H. H. Sanchez-Vives M. V. (2008). Towards a digital body: the virtual arm illusion. Front. Hum. Neurosci. 2:6. 10.3389/neuro.09.006.2008 18958207 PMC2572198

[B47] Slater M. Perez-Marcos D. Ehrsson H. H. Sanchez-Vives M. V. (2009). Inducing illusory ownership of a virtual body. Front. Neurosci. 3, 214–220. 10.3389/neuro.01.029.2009 20011144 PMC2751618

[B48] Thomann A. E. Goettel N. Monsch R. J. Berres M. Jahn T. Steiner L. A.. (2018). The Montreal cognitive assessment: normative data from a german-speaking cohort and comparison with international normative samples. J. Alzheimers Dis. 64, 643–655. 10.3233/jad-180080 29945351 PMC6027948

[B49] Thorne J. D. De Vos M. Viola F. C. Debener S. (2011). Cross-modal phase reset predicts auditory task performance in humans. J. Neurosci. 31, 3853–3861. 10.1523/jneurosci.6176-10.2011 21389240 PMC6622791

[B50] Vuckovic A. Osuagwu B. A. (2013). Using a motor imagery questionnaire to estimate the performance of a Brain-Computer Interface based on object oriented motor imagery. Clin. Neurophysiol. 124, 1586–1589. 10.1016/j.clinph.2013.02.016 23535455

[B51] Wilson A. D. Golonka S. (2013). Embodied cognition is not what you think it is. Front. Psychol. 4:58. 10.3389/fpsyg.2013.00058 23408669 PMC3569617

[B52] Wilson M. (2002). Six views of embodied cognition. Psychon. Bull. Rev. 9, 625–636. 10.3758/bf03196322 12613670

[B53] Yong X. Menon C. (2015). EEG classification of different imaginary movements within the same limb. PLoS One 10:e0121896. 10.1371/journal.pone.0121896 25830611 PMC4382224

[B54] Zich C. Debener S. Schweinitz C. Sterr A. Meekes J. Kranczioch C. (2017). High-intensity chronic stroke motor imagery neurofeedback training at home: three case reports. Clin. EEG Neurosci. 48, 403–412. 10.1177/1550059417717398 28677413

