# Supplementary material for: Exploring Self-Paced Embodiable Neurofeedback for Post-stroke Motor Rehabilitation
Source: Front Hum Neurosci. 2020 Jan 20;13:461. doi: 10.3389/fnhum.2019.00461 (PMC6984194; doi:10.3389/fnhum.2019.00461)

Criteria for CSP filter evaluation

1. Does the signal appear to originate from the sensorimotor areas on sensor level (CSP-Pattern)?

Example 1

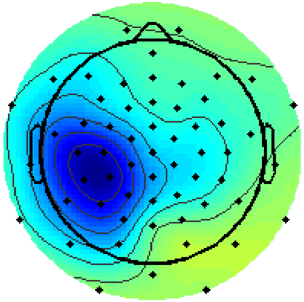

Example 2

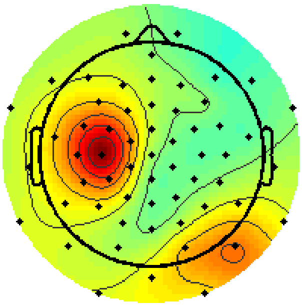

2. Does the signal appear to originate from the sensorimotor areas on source level (CSP-Filter)?

Example 1

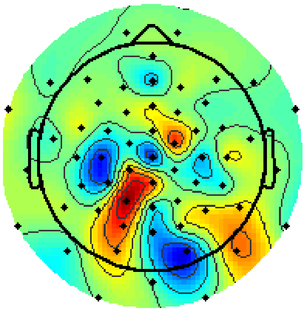

Example 2

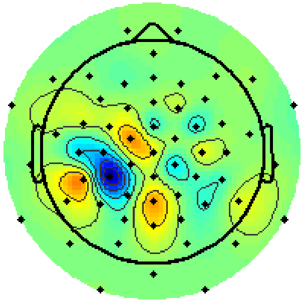

3. Is there a recognizable discriminability in the power value distributions of both trial classes?

Good discriminability

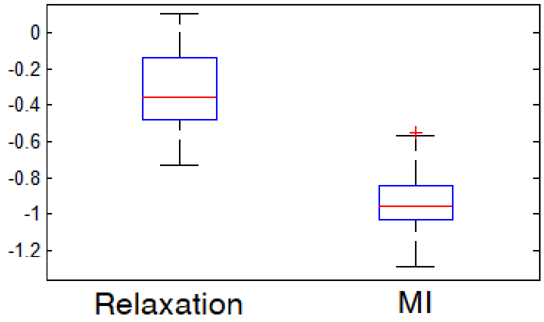

Bad discriminability

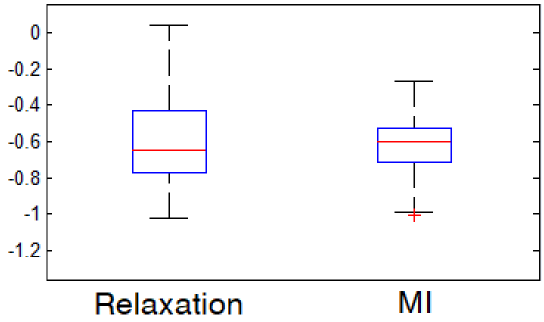

4. Is there a recognizable discriminability on the single-trial time course visualizations of both trial classes?

Good discriminability:

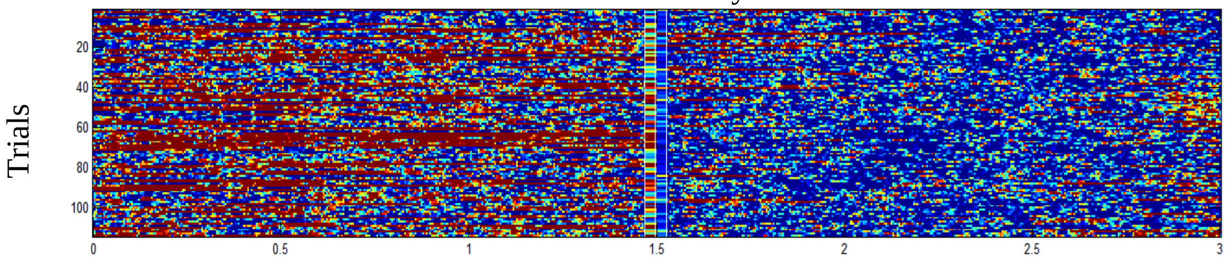

Relaxation MI

Time (sec)

Bad discriminability:

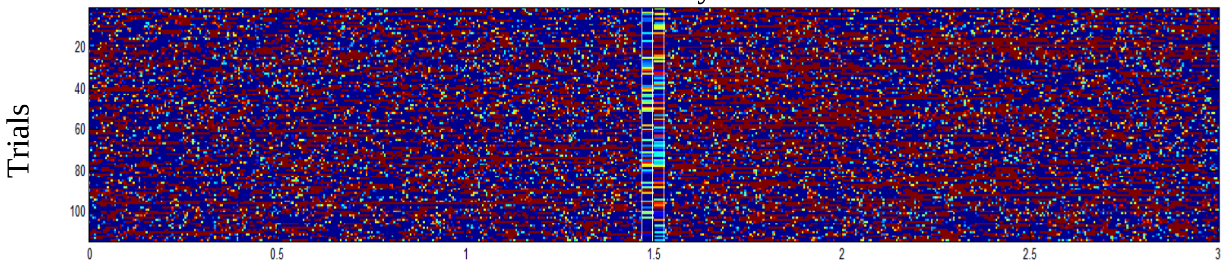

Relaxation MI

Time (sec)

5. Does an event-related desynchronization (ERD) or an event-related synchronization (ERS) occur where expected?

a.) In case that most of the signal appears to originate from the left sensorimotor rhythm: Is there an ERS pattern (i.e., an 8-30 Hz power increase of MI trials, relative to relaxation trials)?

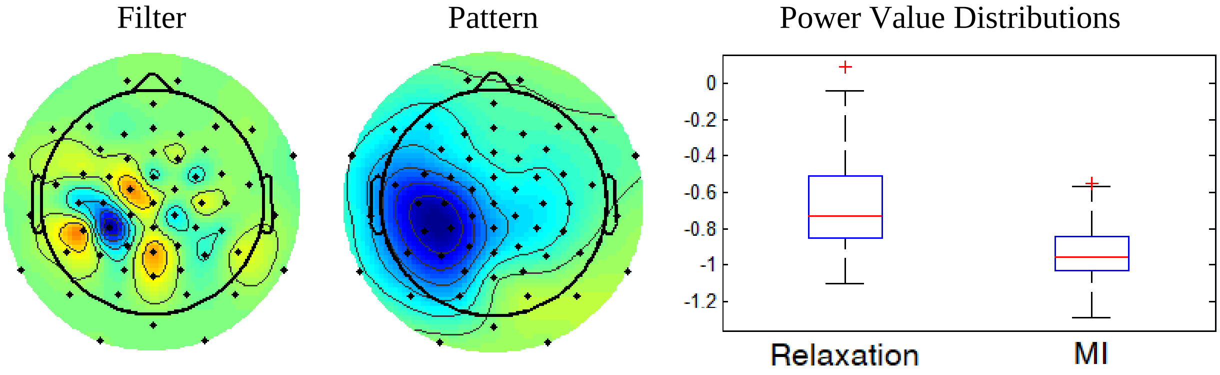

b.) In case that most of the signal appears to originate from the right sensorimotor rhythm: Is there an ERS pattern (i.e., an 8-30 Hz power increase of MI trials, relative to relaxation trials)?

Filter

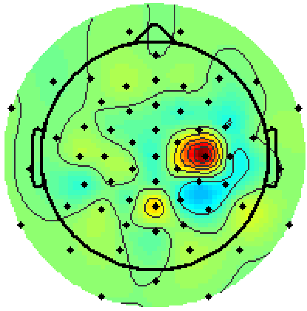

Pattern

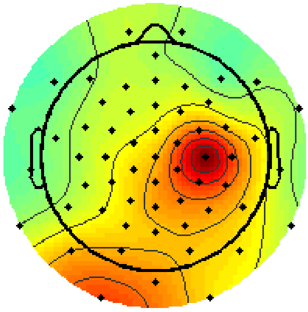

Power Value Distributions

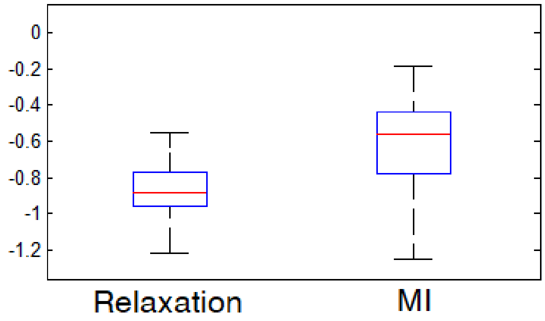

5. Are the power value distributions for both trial classes normally distributed?

Normal Distribution

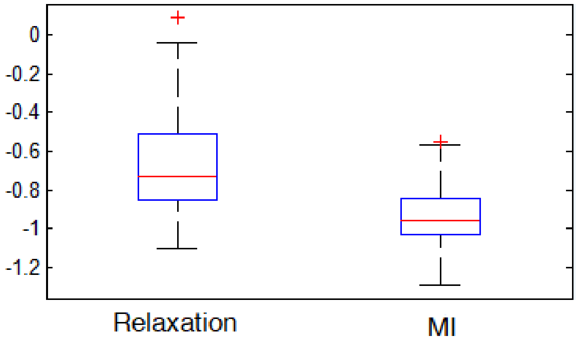

Skewed Distribution

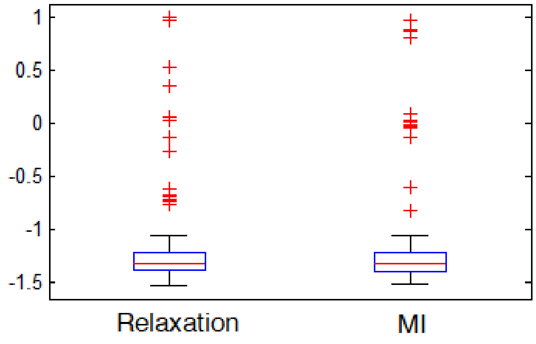

Supplement: Supplementary file 1 [file Data_Sheet_1.pdf]
